# Supplementary material for: Seeking the Membrane-Bound Structure of the Caveolin 8S Complex
Source: J Phys Chem B. 2025 Jul 25;129(31):7932–8. doi: 10.1021/acs.jpcb.5c01585 (PMC12337093; doi:10.1021/acs.jpcb.5c01585)
Supplement: Supplementary file 1 [file jp5c01585_si_001.pdf]

Supporting Information

for

Seeking the Membrane-Bound Structure of the Caveolin 8S Complex

Sayyid Yobhel Vasquez Rodriguez[1] , Themis Lazaridis*[2],

1. CCNY Undergraduate Program, Biology Senior

2. Department of Chemistry,
City College of New York/CUNY, 160 Convent Ave, New York, NY 10031, USA

Graduate Programs in Chemistry, Biochemistry, and Physics,
The Graduate Center, City University of New York,365 Fifth Ave., New York, NY 10016, USA

* Tel. (212) 650-8364 Email: tlazaridis@ccny.cuny.edu

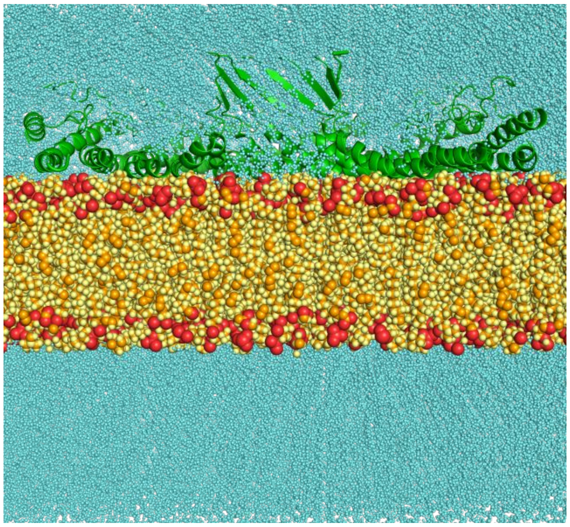

a)

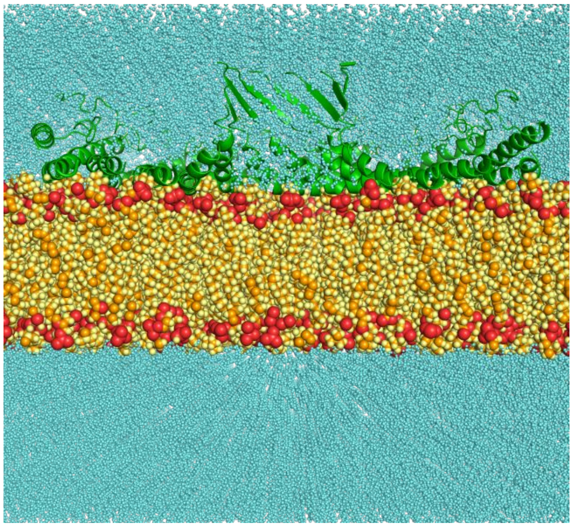

b)

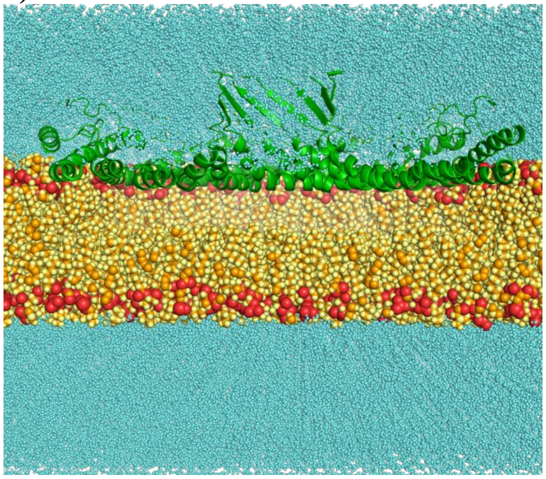

c)

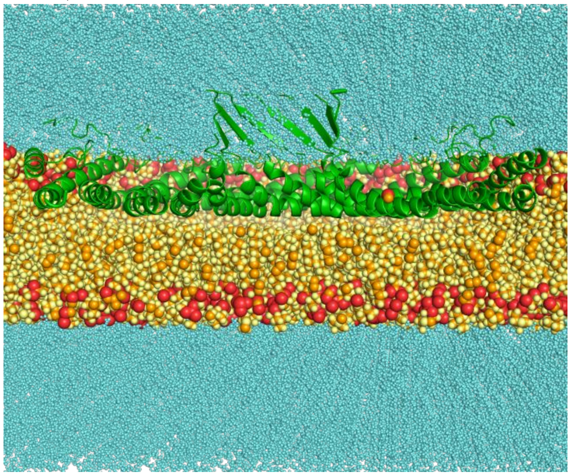

d)

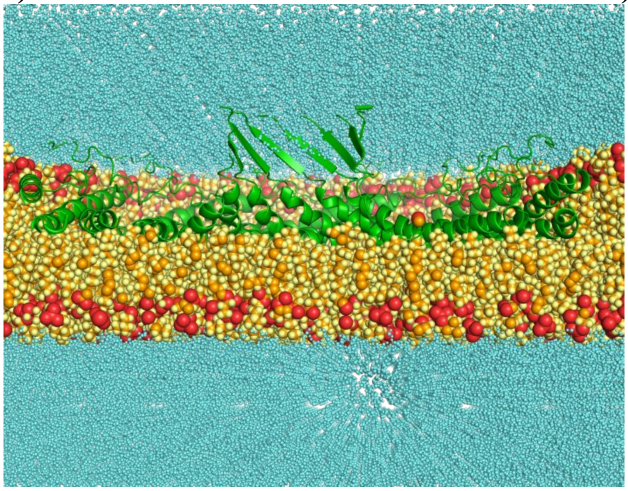

Fig. S1. Starting structures of the bilayer systems. a) 32 Å, b) 30 Å, c) 25 Å, d) 16 Å, e) 10 Å.

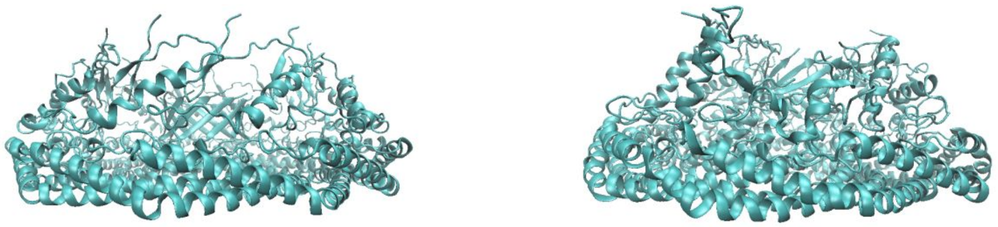

a)

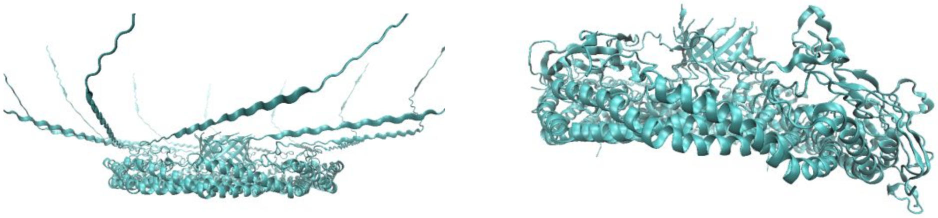

b)

Fig. S2. Implicit solvent (EEF1) simulations of two models of full length caveolin 8S. a) Using the AF2 prediction for residues 1-48, b) Assuming fully extended conformation for residues 1-48. On the left are the starting structures and on the right the final structures after 300 ps simulation.

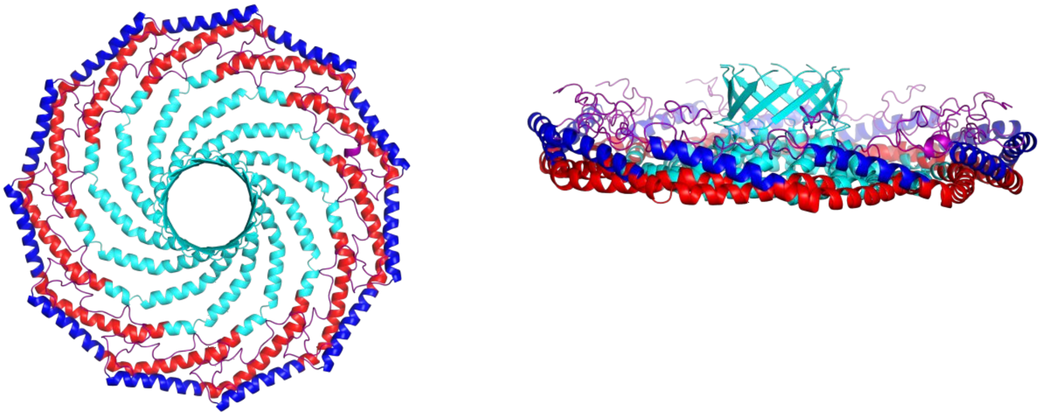

Fig. S3. The sections of CAV-1 are depicted in the following color scheme: purple: residues 49-81 (N-TER), blue: residues 82-101 (Scaffolding Dom), red: residues 102-134 (IMD), cyan: residues 135-178 (C-TER). Left: top view; Right: side view.

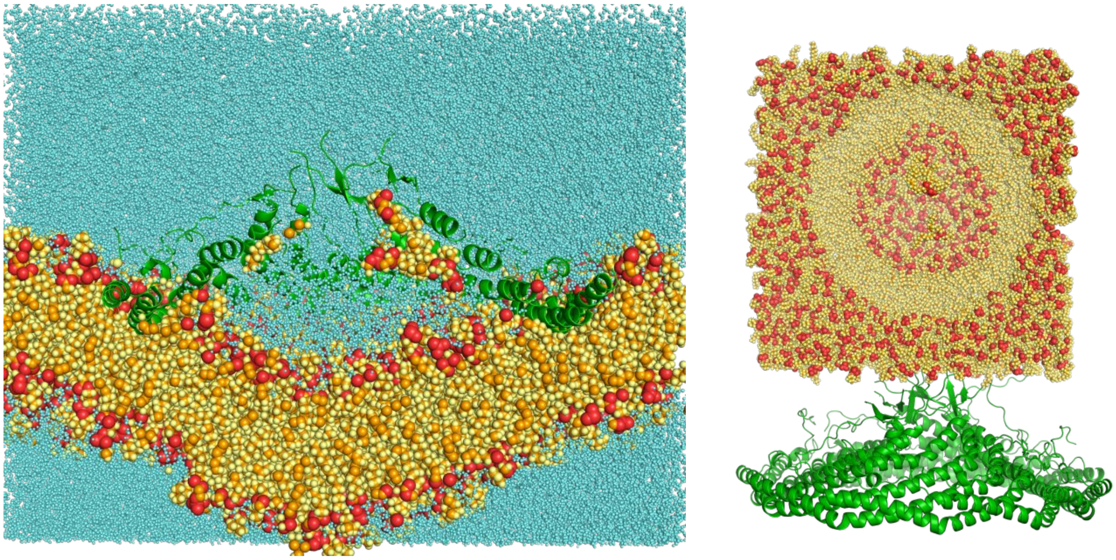

Fig. S4. Replicate of the simulation shown in Fig. 2.

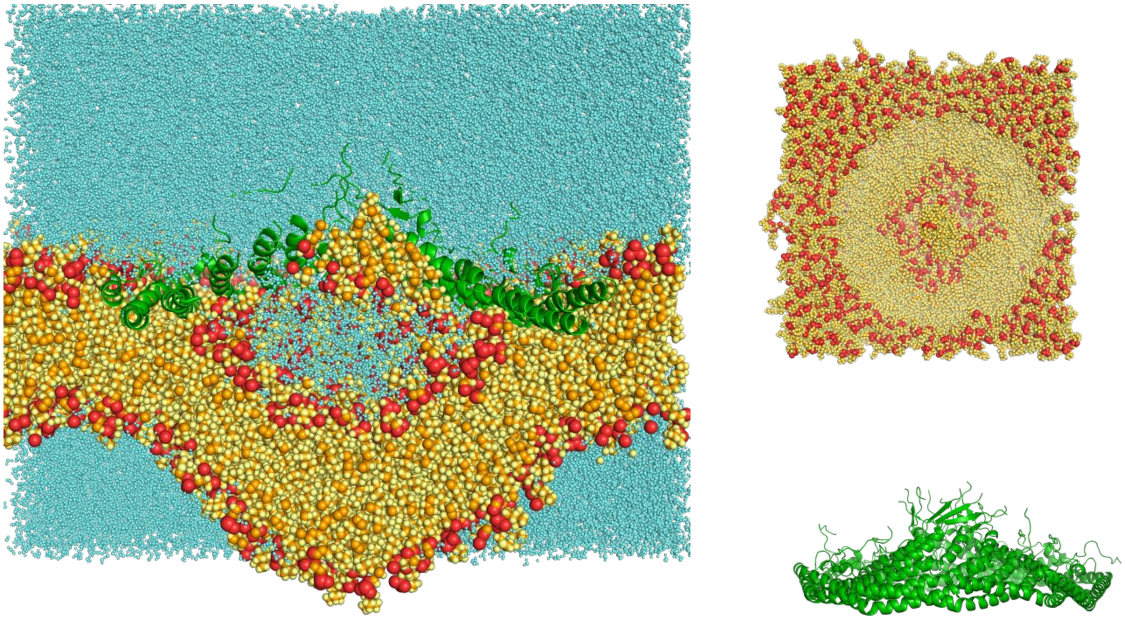

Fig. S5. Replicate of the simulation shown in Fig. 3.

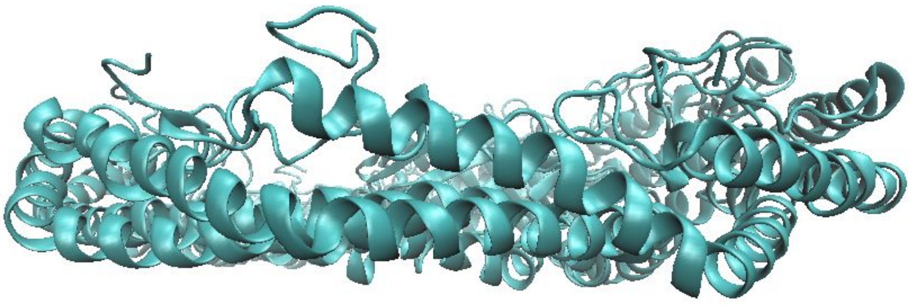

Fig. S6. Conformation of 8S after 9 ns simulation in vacuum.

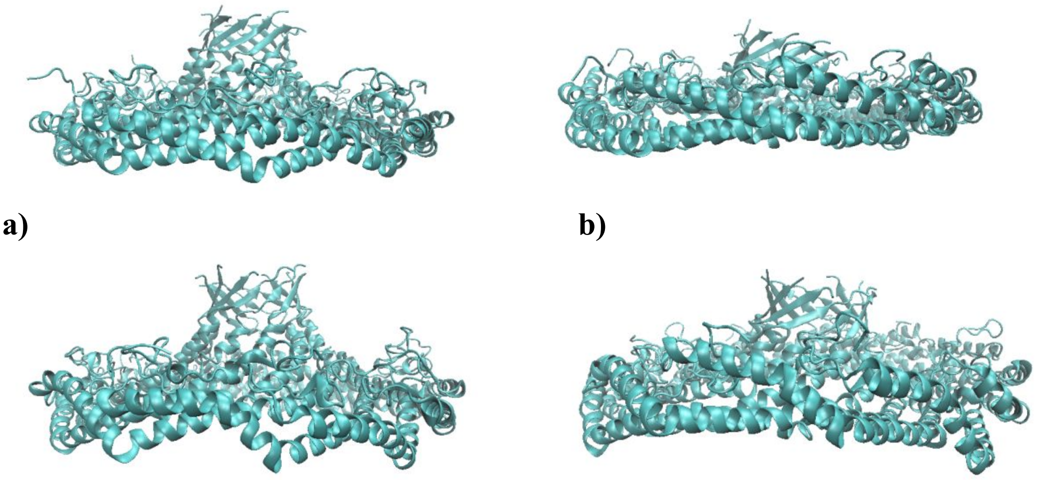

a) **b)**

c) **d)**

Fig. S7. Vacuum simulations starting from conical conformations lead to flatter conformations. a) Structure after 7.35 ns of simulation in explicit water (Ref. 12 main text), spatial extent Δz=57.2 Å b) final structure after 100-ps of simulation of this structure in vacuum, spatial extent Δz=45.7. c) Structure after 100 ps of simulation in implicit water (EEF1), spatial extent Δz=62.9 Å. d) Final structure after 100-ps of simulation of this structure in vacuum, spatial extent Δz=48.9. The spatial extent of the cryo-EM structure is Δz=40 Å.

Table S1. Components of the difference in energy between the energy minimized conformation (b) of Fig. S6 and an early snapshot of the vacuum simulation (which is still conical). Amino-acids are split into four groups: charged (R,K,E,D), polar (S,T,Q,N,G,H), nonpolar (A,V,L,I,C,M,P) and aromatic (W,Y,F). Energies are in kcal/mol. In parenthesis the van der Waals and electrostatic contributions, respectively. The favorable contributions are highlighted in bold.

	ΔE (Conical → Flat)
Total E	-449 (-306,+210)
Intra-charged	**-586** (+32,-590)
Intra-polar	+387 (-31,+433)
Intra-nonpolar	**-762** (-24,-438)
Intra-aromatic	-107 (+11,-72)
Charged-polar	**-454** (-17,-433)
Charged-nonpolar	+62 (-40,101)
Charged-aromatic	+25 (-5,44)
Polar-nonpolar	+701 (-102,810)
Polar-aromatic	**-362** (-24,-341)
Nonpolar-arom	+646 (-46,698)
